# Relationship of prefrontal brain lateralization to optimal cognitive function differs with age

**DOI:** 10.1016/j.neuroimage.2022.119736

**Published:** 2022-11-15

**Authors:** Joseph P. Hennessee, Christina E. Webb, Xi Chen, Kristen M. Kennedy, Gagan S. Wig, Denise C. Park

**Affiliations:** a Center for Vital Longevity, School of Behavioral and Brain Sciences, University of Texas at Dallas, Dallas, TX 75235, USA; b Department of Psychiatry, University of Texas Southwestern Medical Center, Dallas, TX 75390, USA

**Keywords:** Middle-age cognition, Cognitive aging, Brain maintenance, Compensation, Lateralization, Neuroimaging

## Abstract

There is considerable debate about whether additional fMRI-measured activity in the right prefrontal cortex readily observed in older adults represents compensatory activation that enhances cognition or whether maintenance of youthful brain activity best supports cognitive function in late adulthood. To investigate this issue, we tested a large lifespan sample of 461 adults (aged 20–89) and treated degree of left-lateralization in ventrolateral and dorsolateral prefrontal cortex during a semantic judgment fMRI task as an individual differences variable to predict cognition. We found that younger adults were highly left-lateralized, but lateralization did not predict better cognition, whereas higher left-lateralization of prefrontal cortex predicted better cognitive performance in middle-aged adults, providing evidence that left-lateralized, youth-like patterns are optimal in middle age. This relationship was reversed in older adults, with lower laterality scores associated with better cognition. The findings suggest that bilaterality in older adults facilitates cognition, but early manifestation of this pattern during middle age is characteristic of low performers. Implications of these findings for current theories of neurocognitive aging are discussed.

## 1. Introduction

A classic finding in neuroimaging research is that older adults often display more bilateral recruitment of prefrontal cortex (PFC) on tasks that evoke primarily left-lateralized activity in younger adults (Cabeza, 2002; Persson et al., 2006; Reuter-Lorenz and Cappell, 2008). There is some evidence that high-performing older adults show this characteristic bilateral recruitment pattern, which has been interpreted as compensatory brain activity that offsets the negative effect of aging on cognition (Batista et al., 2019; Huang et al., 2012; Reuter-Lorenz and Cappell, 2008; Scheller et al., 2018; Tyler et al., 2010). Recently, Chen et al. (2022) characterized adults as successful or unsuccessful agers based on their decline in processing speed, working memory, reasoning, and episodic memory over approximately four years, finding that older adults who maintained or improved cognitive performance over this interval were more likely than decliners (unsuccessful agers) to show recruitment of additional regions not engaged by young adults in both left and right prefrontal regions. This provided support for a compensation view of frontal activity. However, several recent studies have reported that the absence of this additional PFC activity is associated with superior memory (de Chastelaine et al., 2011; Düzel et al., 2010; Pudas et al., 2018; Vidal-Piñeiro et al., 2019), suggesting that maintenance of youthful brain function with age is a hallmark of better cognitive performance.

The Scaffolding Theory of Aging and Cognition (STAC) suggests that additional prefrontal recruitment with age represents supportive brain circuitry or scaffolding that develops in malleable brain regions and compensates for age-related degradation in brain structure and function that occurs with normal aging (Park and Reuter-Lorenz, 2009; Reuter-Lorenz and Park, 2014). Right ventrolateral (VLPFC) and dorsolateral prefrontal cortex (DLPFC) are strong candidates for compensatory scaffolding as older adults typically show increased activation relative to young in these regions on many cognitive tasks (Cabeza, 2002; Wierenga et al., 2008) and they are implicated in domain-general executive control and working memory processes that are fundamental to cognition (Owen et al., 2005; Simmonds et al., 2008). The DLPFC, in particular, is part of a multi-demand network that helps control complex behavior via linking subcomponents of a task (Duncan, 2013).

It is an important assumption of the STAC model that it is desirable to maintain a youthful brain—both structurally and functionally—and that neural insults (cortical thinning, white matter lesions, amyloid deposition, dedifferentiation of function) that accrue with age represent deviations from the maintenance of a healthy brain that result in compensatory scaffolding. Park and Reuter-Lorenz (2009) note that although this compensatory scaffolded circuitry is beneficial to cognition, it is less efficient than the “intact, finely honed circuitry” of a younger brain, reflecting a maintenance view. The model further suggests that conversion to an aging brain pattern early in life to increased right PFC activity is a marker of poor cognitive function. To assess evidence for this, we treated magnitude of lateralization of VLPFC and DLPFC as an individual differences variable and hypothesized that middle-aged adults who showed evidence of prefrontal bilaterality earlier than others of their age would be lower performers on cognitive tasks compared to those who were maintaining left-lateralization. We also expected that in older adults, bilaterality would be characteristic of successful agers and hypothesized that at older ages, greater bilaterality would be related to better performance, reflecting its compensatory function.

**Fig. 1.**
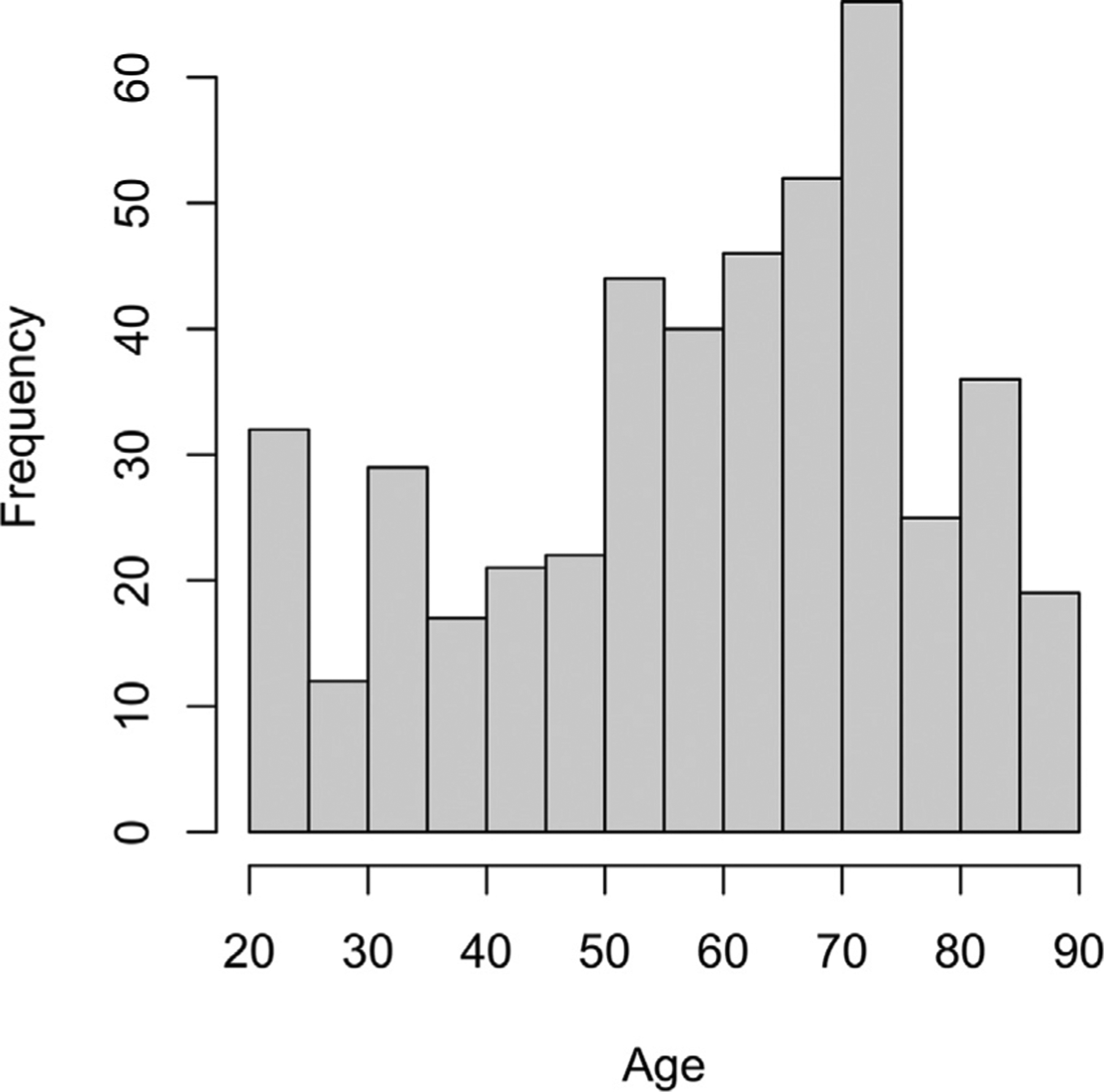
Distribution of participant ages separated in 5-year bins.

To address these questions, we collected measures of functional lateralization in prefrontal cortex from a very large sample of younger (ages 20–39), middle-aged (40–59) and older adults (60–89) who participated in the Dallas Lifespan Brain Study (DLBS, *N* = 461, age distribution in Fig.1). They performed an fMRI semantic word judgement task that has been shown in many studies to show a characteristic left-lateralization patten in younger adults (Binder et al., 2009) with increased right frontal activation in older adults (Cabeza, 2002; Persson et al., 2006; Wierenga et al., 2008); this laterality measure was used as an individual difference variable, along with age, to assess the role of prefrontal lateralization on cognitive performance. Importantly, the large lifespan sample allowed us to determine whether the impact of hemispheric laterality on cognition was different at varying stages of the lifespan under conditions of low or high cognitive demand. Participants made living/non-living judgments about concrete nouns that were either easy (“dog,” “table”) or demanding (“ghost,” “virus”) to classify and we then related magnitude of lateralization in VLPFC and DLPFC to a general measure of cognitive function that combined three core aspects of fluid ability that are interrelated but independent—processing speed, working memory, and reasoning.

Both VLPFC and DLPFC are active on semantic judgment tasks and have also been implicated in compensation in older adults (Gutchess et al., 2005; Persson et al., 2006; Scheller et al., 2018; Tyler et al., 2010), thus our initial analyses used a mask covering both VLPFC and DLPFC. There is evidence that the VLPFC is less sensitive to cognitive demand during semantic judgments (Noonan et al., 2013) and that DLPFC activity is ramped up in younger and older adults when making more difficult judgments requiring greater control (Kennedy et al., 2015; Noonan et al., 2013), although older adults’ ability to increase DLPFC activation for difficult judgments is comparatively lower (Kennedy et al., 2015). Similarly, for working memory tasks, older adults evidence diminished DLPFC activation at high memory loads (Cappell et al., 2010). Thus, we examined activity in these anatomical regions separately and together. We hypothesized that greater bilaterality in both these regions would be advantageous and associated with higher fluid ability in older adults. For middle-aged adults, we expected that youth-like left-lateralization of VLPFC would reflect maintenance and thus serve as a particularly strong predictor of high fluid ability.

Because older adults have been found to have greater right prefrontal recruitment while making unambiguous semantic judgments relative to younger adults (Kennedy et al., 2015; Persson et al., 2006), for our primary analysis we focused on lateralization during unambiguous semantic judgments (low demand condition). We note that a whole brain analysis of the semantic judgment task for a subset of this sample was reported by Kennedy et al. (2015). Here, we focus on lateralization in specific ROI’s and its relationship to general measures of cognition in an effort to determine the optimal neural signature for good cognitive performance, and whether it differs by age.

## 2. Materials and methods

### 2.1. Participants

This study included 461 right-handed participants, ages 20–89, who were screened for neurological and psychiatric disorders, were native English speakers, were well-educated (*M* = 15.78 years of education, *SD* = 2.31) and had an MMSE score of at least 26. All participants were recruited from the community—none were recruited from university human subject pools, although college students were not precluded from participation. There were approximately 50 participants per decade of life from ages 20–50, and older subjects (decades 50–59, 60–69, 70–79 and 80–89) were oversampled. The distribution of participants by age and sex appears in Fig. 1 and Table 1. Participants were recruited using media advertisements and flyers and all gave written informed consent in accordance with the University of Texas Southwestern Medical Center Institutional Review Board.

### 2.2. fMRI task

Participants completed a semantic judgment task for 128 nouns presented in 16 blocks of eight items (half low demand blocks and half high demand) in a Phillips 3T MRI scanner, making a yes/no decision via button box as to whether items were living (right index) or non-living (middle finger). Low demand blocks included eight trials of judgments of words that were concrete objects (dog, table), whereas high demand words were more ambiguous and had characteristics of both living and nonliving things, which required a longer judgment time (ghost, virus; Table 1). Item-order was randomized within blocks and block-order was pseudorandom. Each word was displayed for 2500 ms followed by a 500 ms fixation cross. Total scan time was 7.7 min, including a 6 s fixation interval before the first stimulus block and three 24 s fixation blocks as baseline data. Activation in both demand conditions was contrasted with fixation. Visual stimuli were presented using E-prime (Psychology Software Tools, Pittsburgh, PA, USA) software viewed using a mirror attached to the head coil.

### 2.3. MRI acquisition and structural data processing

All participants were scanned on a single 3T Philips Achieva scanner (Philips Medical Systems, Best, The Netherlands) equipped with an 8-channel head coil. Anatomical data were collected with a T1-weighted MP-RAGE sequence (160 sagittal slices, 1 × 1 × 1 mm^3^ voxels, 204 × 256 × 160 matrix, TR = 8.1 ms, TE = 3.7 ms, flip-angle = 12°). Cortical thickness estimates were derived from the MP-RAGE using Freesurfer ver. 5.3 (Martinos Center for Biomedical Imaging, MA, USA). Extensively trained operators inspected the reconstructed white and gray matter surfaces and performed manual edits when necessary. Functional MRI data were acquired using a T2*-weighted echo-planar imaging sequence with full brain coverage using 43 interleaved axial slices per volume acquired parallel to the AC-PC line (SENSE = 2, 3.4 × 3.4 × 3.5 mm voxels, 64 × 64 × 43 matrix, FOV = 220 × 220 mm, TR = 2 s, TE = 25 ms, flip angle = 80°). Five dummy scans were discarded at the beginning of scanning to allow for T1 stabilization. Images from the scanner were converted to Neuroimaging Informatics Technology Initiative (NIFTI) format using *r2agui*.

### 2.4. fMRI data processing

Data were preprocessed and analyzed using SPM12 (Wellcome center for Human Neuroimaging, London, UK), along with a small number of AFNI (National Institute of Mental Health: Scientific and Statistical Computing Core, MD, USA) and FSL (Wellcome center for Integrative Neuroimaging, Oxford, UK) functions using in-house scripts. Preprocessing began with motion correction: six motion regressors were used as covariates of no interest in SPM during registration. Functional images were normalized to standard MNI space (ICBM152) and resampled into 3 mm^3^ voxels using the T1-weighted structural image for each subject as a coregistered intermediary. The resulting images were smoothed with an isotropic 8 mm FWHM Gaussian kernel. For each subject, neural activity for each task condition (judgements for concrete words, ambiguous judgments, or fixation) was modeled as a block convolved with a canonical hemodynamic response function. An AR(1) model was used to correct for time-series autocorrelations.

### 2.5. Lateralization analysis

A left and right hemisphere cortical mask was created combining VLPFC (pars opercularis, pars triangularis, and pars orbitalis) and DLPFC (rostral and caudal middle frontal) anatomical regions from the Desikan-Killiany atlas (Desikan et al., 2006) along with separate VLPFC and DLPFC masks. For each participant, unweighted laterality indices were computed across the entire mask (VLPFC & DLPFC), and then separately for each of the two regions using the LI-tool, which bootstraps BOLD *t*-values (task – fixation) from each side of a mask iteratively across 20 significance thresholds (Wilke and Lidzba, 2007).^2^ This bootstrapping procedure creates a distribution of estimated laterality indices, and the final index for a subject is their trimmed mean after excluding the distribution’s top and bottom 25%. The index ranges from −1 (completely right-lateralized) to 1 (completely left-lateralized) with 0 indicating no observed lateralization. LI scores between −0.2 and 0.2 are conventionally considered bilateral (Seghier, 2019). These laterality indices were computed from semantic judgments in the low demand condition.

### 2.6. Cognitive measures

We created a measure of fluid ability by averaging the standardized scores for tasks measuring processing speed (Digit Symbol (Wechsler, 1997) and Digit Comparison (adapted from Salthouse and Babcock, 1991)); working memory (Letter-Number Sequencing, Operation Span (Turner and Engle, 1989; Wechsler, 1997)), and reasoning (Educational Testing Service Letter Sets, Raven’s Progressive Matrices (Ekstrom et al., 1976; Raven, 1938)) (Cronbach’s *α* = 0.87). We also created a measure of crystallized ability by computing the average of standardized scores for ETS Advanced Vocabulary I-IV (Ekstrom et al., 1976), Shipley Vocabulary (Zachary and Shipley, 1986), and CANTAB Graded Naming Task (Robbins et al., 1994) (Cronbach’s *α* = 0.84). Episodic memory was the average of standardized scores for Hopkin’s Verbal Learning immediate/delayed recall and recognition and CANTAB verbal recognition memory immediate recall (Brandt, 1991; Robbins et al., 1994) (Cronbach’s *α* = 0.79). Missing data were imputed using expectation-maximization.

Large-scale studies have shown that fluid ability declines across the lifespan (Park et al., 2002; Salthouse et al., 2008) and that it relies on multi-modal associative processing in lateral prefrontal and parietal cortices (Basten et al., 2015; Jung and Haier, 2007). We also note that, in contrast to fluid ability, crystallized ability indexes stored knowledge, reflecting the impact of experience on cognitive ability, and is typically measured by breadth of vocabulary (Diehl et al., 1995; Horn and Cattell, 1967) and increases modestly with age (Park et al., 2002; Salthouse et al., 2008). It is primarily a measure of knowledge, and thus we controlled for this variable, due to our focus on fluid abilities rather than stored content.

### 2.7. Statistical analysis

Our statistical analyses were conducted in six key steps and were computed in R 3.6.2 (R Core Team, 2019). As the first step in our analysis plan, we wanted to ensure that we observed expected effects of age for fluid ability, crystallized ability, and laterality in the VLPFC/DLPFC mask. Our expectation was that fluid ability and laterality would show a decrease with age, whereas crystallized ability would be slightly elevated in older age. Three one-way ANOVAs using an unweighted means analysis were computed using the *aov* function (Type-III SS) with age group (young, middle-aged, old) as the between-groups variable and laterality, fluid ability, or crystallized ability as the outcome variable. Post hoc age group comparisons were made using Tukey’s HSD test (*TukeyHSD*).

Second, once we verified that these usual functions of age were observed in the sample, we then conducted a univariate analysis of BOLD signal during semantic judgments (task–fixation) in the low and high demand conditions to determine that there was sufficient activity in the DLPFC and VLPFC to test our hypotheses.

Third, we wanted to determine initially whether the relationship of laterality to fluid ability differed as a function of age group. To assess this, we focused initially on evidence for an age x laterality interaction in the low demand condition using a multiple regression model. In this initial analysis, we treated age as a categorical variable (young, middle-aged, old), as this allowed us to test *a priori* predictions about age group. We also selected a region from the occipital cortex (pericalcarine cortex) to repeat the laterality analyses on a region that we did not expect to show the predicted pattern or results, in an effort to provide some evidence for the specificity of laterality effects to the VLPFC and DLPFC. All regressions were computed using the *lm* function with simple slopes estimated from the *interactions* package ver. 1.1.1 (Long, 2019). Predictors included age group and laterality computed from the combined VLPFC/DLPFC mask, with fluid ability as the outcome measure. In all of the analyses, continuous variables—crystallized ability, laterality, and fluid ability—were standardized prior to model entry, and sex and crystallized ability were entered as covariates unless otherwise noted.

In a fourth step in the analysis, we took a more granular approach to the VLPFC/DLPFC ROI. We separately assessed whether the patterns of finding observed in the combined DLPFC and VLPFC regions were replicated for high demand and low demand items and for the individual regions of the DLPFC and VLPFC. Thus, the following four new laterality indices were computed: VLPFC-low demand, VLPFC-high demand, DLPFC-low demand, and DLPFC-high demand. The regression model used to predict fluid ability described in step 3 was repeated four times with each iteration using one of the four laterality scores described above. Then, to determine whether the relationship between laterality and fluid ability in each age group differed significantly by region (VLPFC vs. DLPFC) or semantic judgment demand, Pearson correlation coefficients were compared using Steiger’s *z*-test (Steiger, 1980) using standardized residuals.

Fifth, to better understand how age moderates the association between laterality and fluid ability continuously across the lifespan, the regression model used to predict fluid ability in step 2 was repeated, but age was treated as a continuous variable with non-linear effects modeled using quadratic and cubic terms. Regions of significance were derived for the age variable using the Johnson-Neyman procedure in the *interactions* package.

Finally, in a sixth step, additional multiple regressions were conducted that examined: (a) whether the pattern of results observed for fluid ability was reliable for the component tasks comprising fluid ability, (b) whether findings would be preserved if judgment RT was used as a control variable, and (c) whether prefrontal cortical thickness–as a measure of brain degradation—would predict bilateral frontal recruitment, particularly in middle-age, as the STAC model specifies (Park and Reuter-Lorenz, 2009).

## 3. Results

### 3.1. Age effects for prefrontal lateralization, in-scanner task performance, and measures of cognition

A between-subjects ANOVA was used to determine whether lateralization in the combined VLPFC/DLPFC mask showed expected differences across the three age groups (young, middle-aged, old) with *post hoc* comparisons made using Tukey’s HSD test (Table 1). A significant effect of age group was observed, *F*(2458) = 6.01, *p* = .003, $\eta_{p}^{2}=\;0.03$. All groups showed an activation bias toward left prefrontal cortex, but the degree of left-lateralization in the low demand condition decreased across the three age groups (Fig. 4b), and significantly differed between younger adults (*M* = 0.43, *SD* = 0.37) and older adults (*M* = 0.28, *SD* = 0.40), likely due to increased recruitment in right prefrontal for older adults (Fig. 3, Table 2). For the corresponding in-scanner semantic judgement task, classification accuracy was at ceiling for low demand trials (*M* = 0.97, SD = *0*.06), and a main effect of age group indicated that older participants had longer median RTs than younger participants for both low demand, *F*(2, 458) = 15.17, *p* < .001, and high demand trials, *F*(2, 458) = 6.65, *p* = .001.

**Fig. 2.**
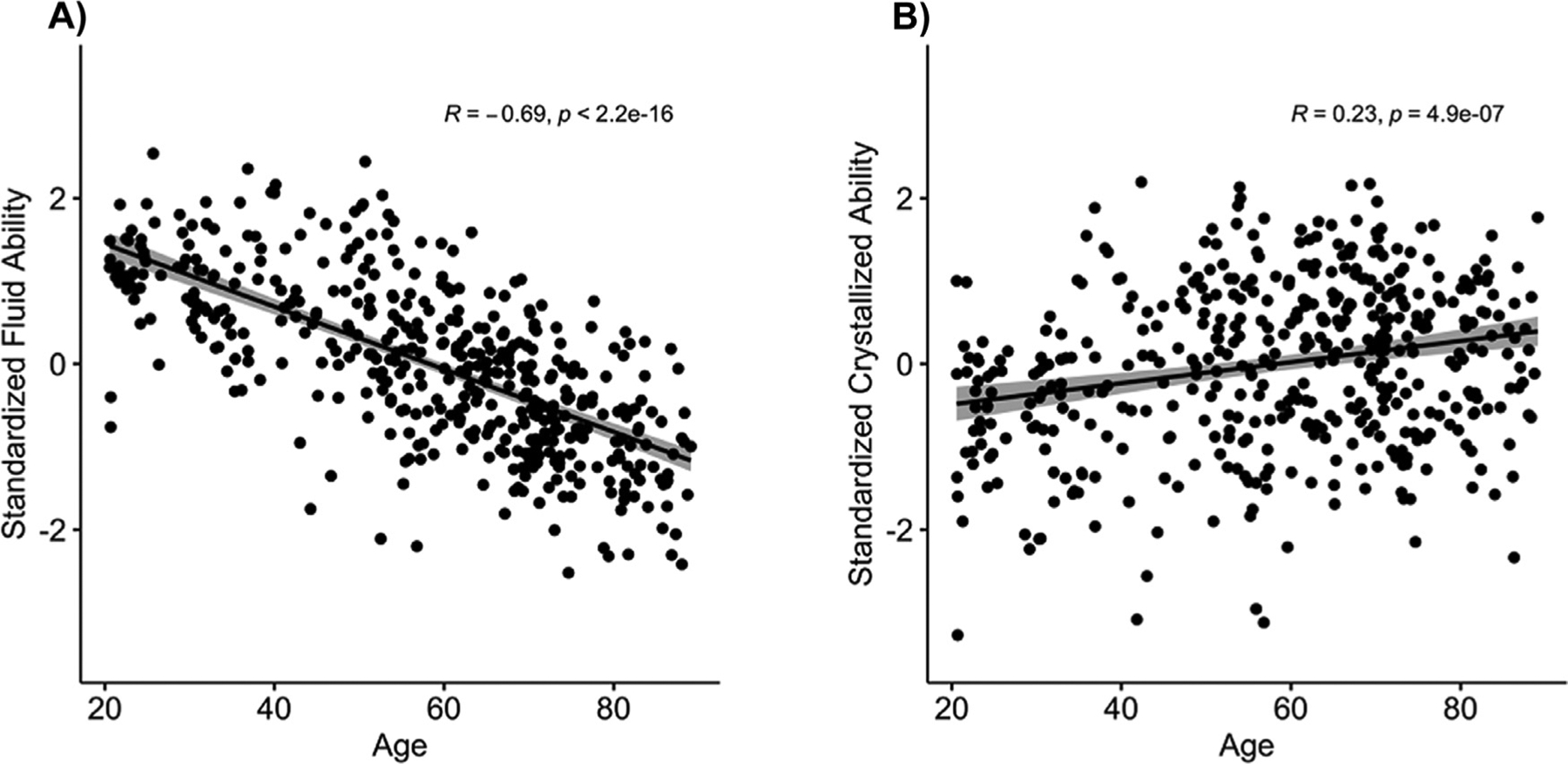
Association between age and A) fluid ability, and B) crystallized ability including linear trendlines.

The one-way ANOVA on fluid ability yielded a main effect of age, *F*(2458) = 145.55, *p* < .001, $\eta_{p}^{2}=\;0.39$, that occurred as a result of a significant decrease in performance across the three age groups, with younger adults (*M* = 0.98, *SD* = 0.63) generally showing better ability than middle-aged adults (*M* = 0.37, *SD* = 0.92), and middle-aged adults evidencing better ability than older adults (*M* = −0.55, *SD* = 0.76, also see Fig. 2). This pattern of findings was also evidenced across the three subcomponents of fluid ability: processing speed, fluid reasoning, and working memory. An analysis of crystallized ability yielded an age main effect *F*(2458) = 16.94, *p* < .001, $\eta_{p}^{2}=\;0.07$, that was significant because vocabulary was higher in older participants. The lowest scores were in younger adults (*M* = −0.50, *SD* = 0.92), and middle-aged (*M* = −0.02, *SD* = 1.11) and older adults (*M* = 0.19, *SD* = 0.90) demonstrated increasingly higher scores. All of these initial results for laterality, fluid ability, and crystalized ability showed expected effects of age observed both in our previous work (Park et al., 202) and by others (Salthouse et al., 2008; Wierenga et al., 2008). The results are displayed in Figs. 2 and 4b. We note that there was a significant, though relatively modest, correlation between crystallized and fluid ability scores in the full sample, *r* = 0.22, *p* = .001, suggesting some overlap between the two constructs, but the two measures were deemed sufficiently independent of one another to be treated separately.

### 3.2. Univariate analysis

**Fig. 3.**
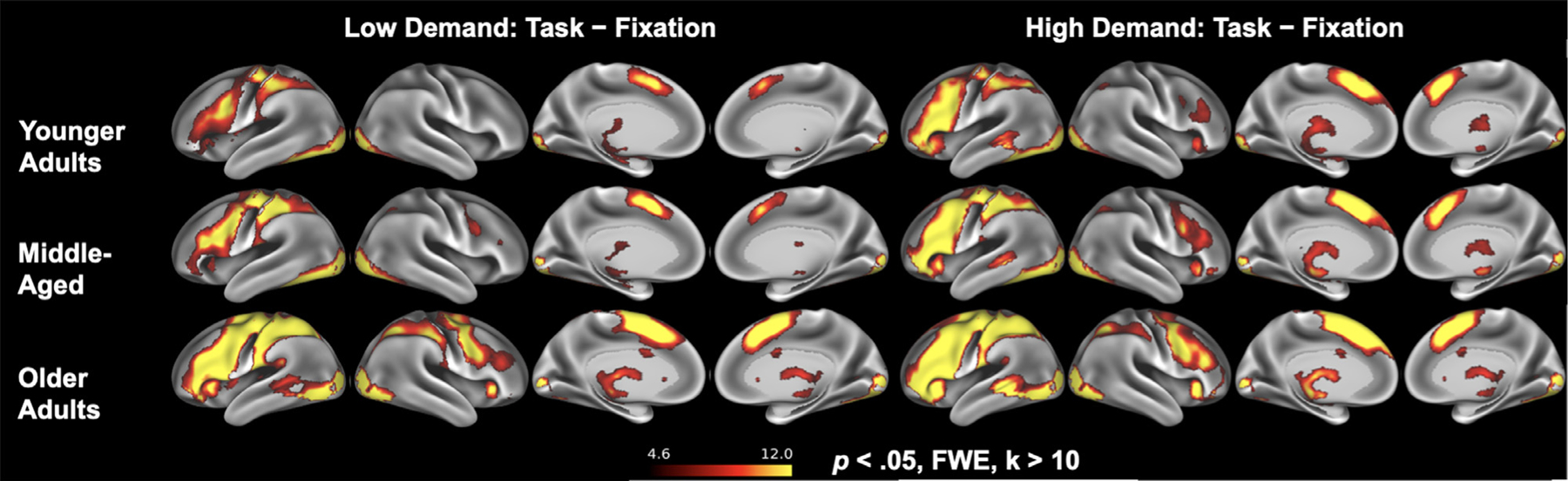
Activation *t*-value for low demand semantic judgments (left) and for high demand judgments (right) in younger (ages 20–39, *n* = 90), middle-aged (ages 40–59, *n* = 127), and older adults (ages 60–89, *n* = 244). Activation in lateral prefrontal cortex was highly left-lateralized in younger adults but showed increasing bilaterality in older age groups and with higher task demand.

In order to determine that the regions we selected for analysis were activated, we conducted two univariate analyses based on the contrast of high demand items and low demand items minus fixation. Results are presented in Fig. 3 and Table 2.

### 3.3. Association between prefrontal lateralization and fluid ability across age groups

**Fig. 4.**
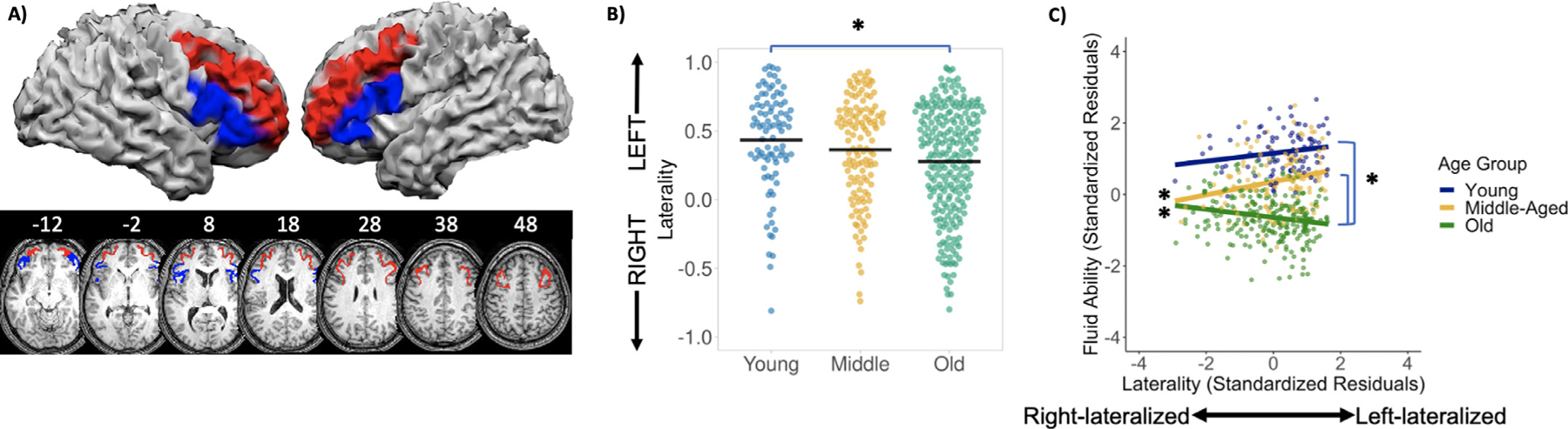
(A) Ventrolateral (blue) and dorsolateral (red) portions of the prefrontal mask overlaid on a representative subject’s normalized anatomical image. (B) Plots of prefrontal laterality scores (low demand − fixation) by age group (young: 20–39, middle-aged: 40–59, old: 60–89), *p < .05 for Tukey’s HSD. (C) Moderation effect of age group on the association between prefrontal lateralization (low demand − fixation) and fluid ability. Left asterisks indicate significant simple slopes and right asterisks indicate significant slope differences.

The primary analysis focused on determining whether the association between laterality in the combined VLPFC/DLPFC mask and cognitive performance differed as a function of age group. A multiple regression predicting fluid ability was computed and included sex and crystallized ability as covariates, and age group, lateralization, and age group x lateralization as the effects of interest (Table 3). The overall model was significant, *F*(7, 453) = 84.25, *p* < .001, *R*^2^ = 0.57, and moreover, a significant age group x lateralization interaction was observed, *F*(2, 453) = 9.28, *p* < .001.^3^ As depicted in Fig. 4, this interaction occurred because greater left-lateralization was related to higher fluid ability in middle-age (*β* = 0.19, *p* = .003), whereas for older adults, better performers on fluid ability exhibited decreased lateralization scores (*β* = −.11, *p* = .006). The effect of lateralization in younger adults was not statistically significant, *β* = 0.11, *p* = .141. Hence, stronger left-lateralization of PFC was related to increasing fluid ability in middle-age, a pattern consistent with brain maintenance. In contrast, older adults with better fluid ability showed more bilateral frontal recruitment. When directly comparing age groups, the effect of laterality significantly differed between middle-aged and older adults (*β* = −.30, *p* < .001, Table 3) and between younger and older adults (*β* = −.22, *p* = .009), but did not differ between younger and middle-aged adults (*β* = −.07, *p* = .446).

**Fig. 5.**
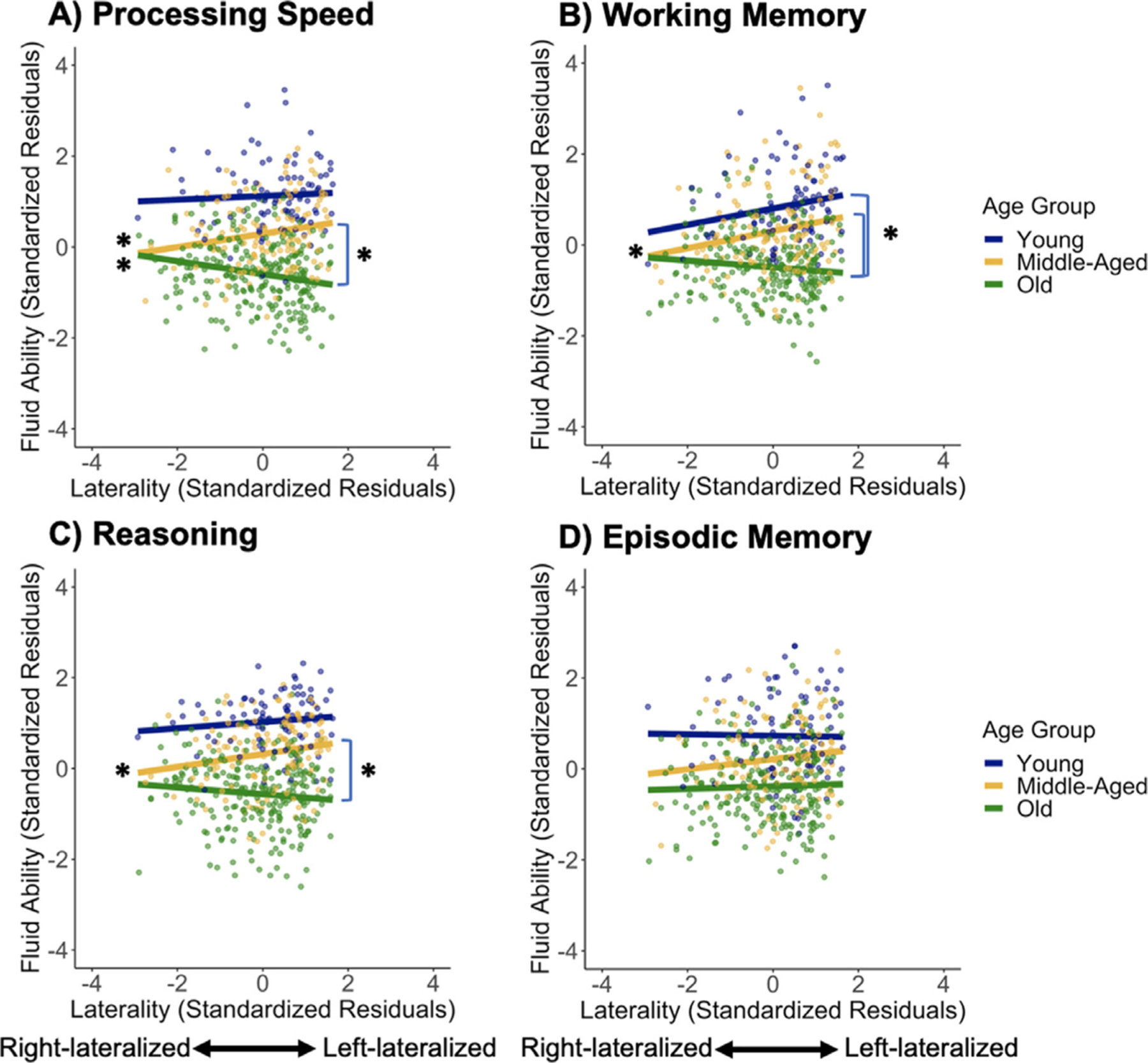
Moderation effect of age group on the association between prefrontal lateralization (low demand – fixation) and the outcome variables: (A) processing speed, (B) working memory, (C) reasoning, and (D) episodic memory. Left asterisks indicate significant simple slopes and right asterisks indicate significant slope differences.

Additional analyses indicated that these effects of laterality persisted when controlling for semantic judgment RTs, with a significant age group x lateralization interaction observed, *F*(8, 452) = 6.68, *p* = .001. There, greater left-lateralization was significantly related to higher fluid ability in middle-aged adults (*β* = 0.17, *p* = .004), bilaterality showed a trend with higher performance in older adults (*β* = −.07, *p* = .079), and laterality was unrelated to performance in younger adults (*β* = 0.11, *p* = .119). These effects also appear to be specific to prefrontal cortex as lateralization of pericalcarine cortex, an active region selected to be a control region, was unrelated to fluid ability with both the main effect of pericalcarine laterality, *F*(1, 453) = 0.36, *p* = .549, and the age group x laterality interaction, *F*(2, 453) = 0.35, *p* = .707, not significant. Finally, analyses for subcomponents of fluid ability (processing speed, working memory, and reasoning) yielded similar patterns (Fig. 5), although laterality was not significantly related to episodic memory. More specifically, after Bonferroni correction for the four tests (*α* = 0.0125), as in our fluid ability analysis, the age group x laterality (VLPFC/DLPFC) interaction was significant for processing speed [*F*(1, 453) = 6.73, *p* = .001] and working memory, [*F*(1, 453) = 5.60, *p* = .004], with a trend for reasoning, *F*(2, 453) = 3.90, *p* = .020, but not significant for episodic memory, *F*(1, 455) = 0.61, *p* = .546.

### 3.4. The association between lateralization and fluid ability by prefrontal subregions and task demand

To more precisely localize these lateralization effects and determine whether they would be affected by increased task demands, laterality indices were computed for VLPFC and DLPFC for both the low and high demand semantic judgment conditions (Table 1). A mixed measures ANOVA was computed to determine whether laterality indices differed as a function of age group, judgment demand (low or high), or region (VLPFC or DLPFC). A significant main effect of region was observed, *F*(1458) = 257.01, *p* < .001, $\eta_{p}^{2}=\;0.36$, as participants were substantially more left-lateralized in VLPFC (*M* = 0.50, *SD* = 0.37) than in DLPFC (*M* = 0.24, *SD* = 0.43). The age group x region x demand interaction was not significant, *F*(2458) = 1.49, *p* = .226, $\eta_{p}^{2}=\;0.01$. The only significant interaction here was between demand and age group, *F*(2458) = 5.85, *p* = .003, $\eta_{p}^{2}=\;0.03$; post hoc *t*-tests for both masks revealed that this interaction occurred because younger adults showed no significant differences in laterality by demand (all *p*’s > 0.2), whereas both middle-aged and older adults showed greater left-lateralization for the high demand condition relative to the low demand condition (all *p*’s < 0.001).

**Fig. 6.**
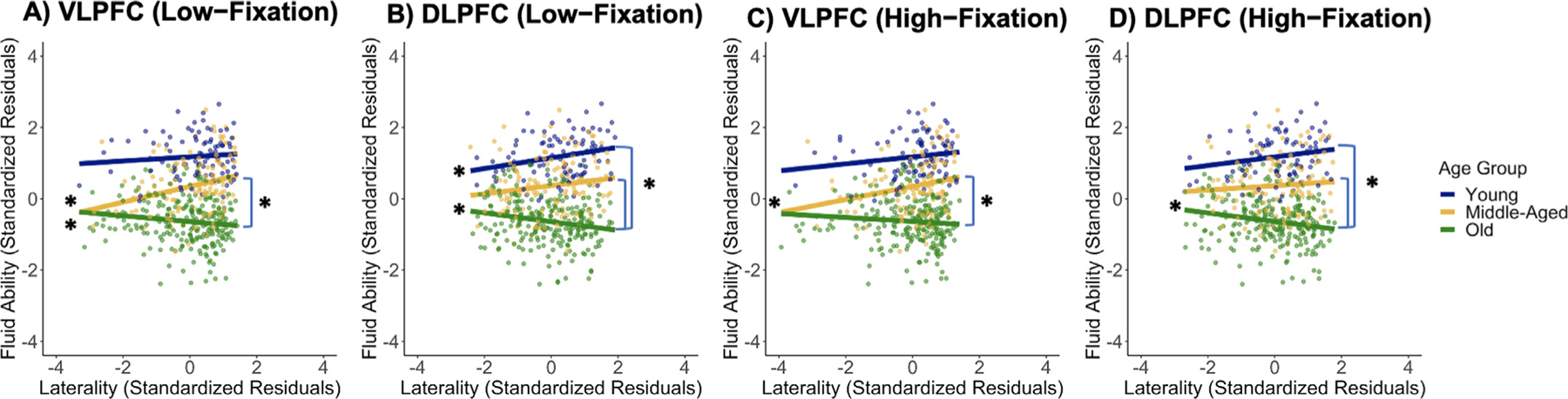
Moderation effect of age group on the association between prefrontal lateralization and fluid ability when lateralization was computed in the VLPFC and DLPFC during low demand semantic judgments (A & B) and during high demand semantic judgments (C & D). Left asterisks indicate significant simple slopes and right asterisks indicate significant slope differences. Abbreviations: DLPFC, dorsolateral prefrontal cortex; VLPFC, ventrolateral prefrontal cortex.

Laterality scores from the four conditions produced by the region x demand interaction were then used to predict fluid ability via regressions testing the age group, laterality, and age group x laterality effects with a Bonferroni correction for the four regressions (*α* = 0.0125, Fig. 6). For low demand semantic judgments/VLPFC the age group x laterality interaction was significant, *F*(2, 453) = 8.15, *p* < .001 and it was also significant for low demand judgments/DLPFC, *F*(2, 453) = 8.20, *p* < .001. For high demand judgments/VLPFC, the age group x laterality interaction was again significant, *F*(2, 453) = 5.36, *p* = .005, and also for high demand judgements/DLPFC, *F*(2, 453) = 5.52, *p* = .004. As seen in Fig. 6, the age group x laterality interaction in predicting fluid ability was highly similar to that seen in our primary model (Fig. 4c) regardless of whether laterality was estimated only from one of the two regions or from the high demand condition. Next, to determine whether the above associations between laterality and fluid ability differed as a function of region (VLPFC or DLPFC) or semantic judgment demand, correlations controlled for sex and crystallized ability were computed in each age group and compared using Steiger’s *z*-test. No differences were observed based on region (all *p*’s > 0.05) or task demand (all *p*’s > 0.14), which indicates that differences in the association between laterality and fluid ability based on region (VLPFC vs. DLPFC) or semantic judgment demand were fairly limited.

### 3.5. Effect of age as a continuous moderator

**Fig. 7.**
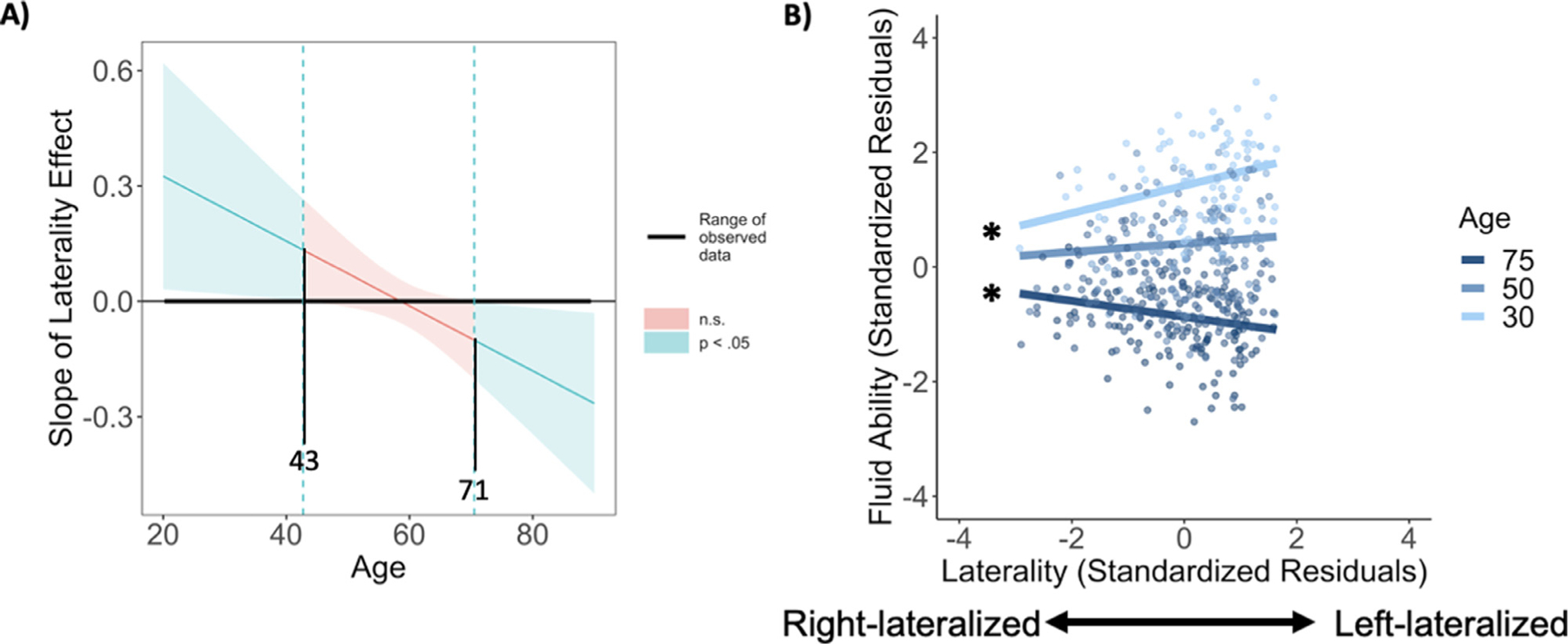
(A) Conditional effect of prefrontal laterality (low demand − fixation) on fluid ability at different values of the continuous moderator variable age. Confidence bands estimated using the Johnson-Neyman procedure indicate the age ranges at which laterality had a significant, or non-significant relationship to fluid ability. For those under age ~43, stronger left-lateralization was significantly related to higher fluid ability (positive laterality slope), whereas decreased left-lateralization (greater bilaterality) was related to better fluid ability in those over ~71 (negative laterality slope). (B) Moderation effect of continuous age on the relationship between prefrontal laterality (low demand − fixation) and fluid ability. Left asterisks indicate significant simple slopes.

In all of the previous analyses, we treated age as a categorical variable due to *a priori* hypotheses regarding differences among age groups. To further understand the effect of age, we conducted a second set of regressions with age treated as a continuous variable, allowing us to examine both linear and non-linear effects of age. The initial regression included age_linear_, age_quadratic_, and age_cubic_ terms, sex, crystallized ability, and laterality from the combined VLPFC/DLPFC mask during low demand judgments. We note that the reversal of the laterality-fluid ability relationship as age increases may suggest some degree of a cubic trend, and indeed model fit was significantly improved when adding the cubic age term, *F*(2451) = 2.78, *p* = .027, but was not significantly improved when adding the quadratic age term, *F*(2, 451) = 2.28, *p* = .104, as opposed to only examining linear effects of age. The final model significantly predicted fluid ability, *F*(9, 451) = 91.25, *p* < .001, *R*^2^ = 0.65 (Fig. 6, Table 3), with only the age_linear_ by prefrontal laterality interaction significant (*α* = −.15, *p* = .025). The Johnson-Neyman procedure and a simple slopes analysis were used to assess the effect of laterality on fluid ability at different ages (Preacher et al., 2006). Congruent with the previous analysis, bilaterality was associated with better performance for those who were age ~71 or greater, as shown in Fig. 7a. Diverging somewhat from the categorical age model, Fig. 7a illustrates that adults below age ~43 had better fluid ability with stronger left-lateralization, but that the relationship between laterality and fluid ability was not significant in adults between ages ~44–70. A simple slopes analysis was conducted to link the continuous model to the categorical model by using the midpoints of the three categorical age groups (ages 30, 50, and 75) reported earlier. This approach yielded a significant effect of laterality at age 30 (*β* = 0.24, *p* = .034) with left-lateralization associated with higher fluid ability. A positive relationship between laterality and fluid ability was only a trend at age 50 (*β* = 0.07, *p* = .105), and at age 75 the relationship reversed as those with greater bilaterality evidenced higher fluid ability (*β* = −.14, *p* = .036).

### 3.6. Semantic judgment response time and prefrontal lateralization

When repeating our primary models using age group and laterality as predictors and sex and crystallized ability as covariates to predict judgments RTs, we observed, consistent with a compensatory account, that older adults with more bilateral prefrontal recruitment showed significantly shorter RTs for both the low demand (*β* = 0.19, *p* = .001) and high demand conditions (*β* = 0.17, *p* = .006). We note that low demand RTs were negatively correlated with fluid ability (*r* = −.46, *p* < .001) and crystallized ability (*r* = −.20, *p* < .001), and high demand RTs were also negatively correlated with fluid ability (*r* = −.23, *p* < .001), but not crystallized ability (*r* < 0.01, *p* = .979).

### 3.7. Associations between prefrontal cortical thickness and lateralization

Cortical thickness values for our primary left and right prefrontal (VLPFC/DLPFC) ROI are listed in Table 1, and as expected, thickness was significantly related to age group for both the left ROI, *F*(2, 460) = 71.34, *p* < .001, and right ROI, *F*(2, 460) = 90.53, *p* < .001, as older participants had thinner cortex. To investigate whether lower thickness was related to greater prefrontal bilaterality during semantic retrieval, presumably as an attempt to compensate, thickness values were first split into tertiles across the full sample to avoid non-linear effects of modest thickness differences. A multiple regression was conducted predicting prefrontal laterality (low demand condition) using age group and left prefrontal thickness tertile as predictors, and age, sex, and crystallized ability as covariates. Importantly, a significant age group by thickness tertile interaction was observed, *F*(2, 453) = 4.28, *p* = .014. In middle-aged adults, being in a lower left prefrontal thickness tertile was related to greater bilaterality (*ß* = 0.26, *p* = .027), though thickness tertile was not significantly related to laterality in younger (*ß* = −.37, *p* = .068) or older adults (*ß* = −.05, *p* = .578).

## 4. Discussion

The present study confirmed our hypothesis that, during unambiguous semantic judgments (low demand condition), patterns of functional lateralization in prefrontal cortex (VLPFC and DLPFC) associated with optimal cognition differ as a function of age. Analyses from the categorical age model revealed that middle-aged adults showed a consistently positive association between fluid ability and more left-lateralized activation patterns in prefrontal cortex. In contrast, older adults evidenced better fluid ability, and shorter semantic judgement RTs, with more bilateral, rather than left-lateralized, prefrontal recruitment. Finally, although younger adults were highly left-lateralized on the semantic judgment task, as reported in other studies (Persson et al., 2006; Wierenga et al., 2008), their magnitude of left-lateralization in PFC was unrelated to fluid ability. Similar, but not identical, findings were observed when age was treated as a continuous variable, as discussed below. Other findings included evidence that, as expected, younger adults were more left-lateralized than older adults, whereas the degree of lateralization in middle-age fell between these two extremes. Also, consistent with prior work (Park et al., 2002; Salthouse et al., 2008, 2014), fluid ability scores decreased with age, and crystallized ability scores modestly increased across the lifespan. Finally, although laterality indices derived from judgments of ambiguous items (high demand condition) did not significantly vary as a function of age as all age groups displayed fairly strong left-lateralization, relationships between these lateralization indices and fluid ability did not differ markedly from what was observed for the low demand condition.

When lateralization was examined across the three age groups separately for VLPFC and DLPFC, and for low and high demand semantic judgments, we observed that VLPFC showed considerably greater left-lateralization than did DLPFC, and that DLPFC recruitment was fairly bilateral, particularly in middle-aged and older adults. This was consistent with prior work showing that VLPFC has particularly strong left hemispheric dominance during semantic judgments (Binder et al., 2009). Additionally, the effect of judgment task demands on laterality varied by age group, such that middle-aged and older adults showed increased left-lateralization when making more difficult judgments, whereas younger adults did not. This finding illustrates that prefrontal lateralization during semantic judgments differs in a complex way across the lifespan, as prefrontal recruitment becomes more bilateral with age (Persson et al., 2006; Wierenga et al., 2008), but when middle-aged and older adults make more difficult judgments requiring additional semantic control somewhat greater reliance may be placed on primary task regions in left prefrontal. Although we had predicted that relationships between lateralization and fluid ability may differ between these two regions and judgment difficulties, no significant differences were observed here. This finding was somewhat unexpected as DLPFC has been shown to play a particularly important role in completing challenging cognitive tasks (Duncan, 2013; Reuter-Lorenz and Cappell, 2008). Instead, these results suggest that, for semantic judgments, VLPFC and DLPFC play complimentary roles in compensation in older adults and maintenance in middle-age, and that these effects do not appear to depend strongly on judgment demands.

We treated age as a continuous variable to further confirm these relationships, and the pattern of findings was fairly similar, but with added nuance regarding the age x laterality interaction. Results of a Johnson-Neyman analysis indicated that strong left-lateralization during semantic judgments was related to higher fluid ability from young adulthood through early middle age (~43 yrs.), and greater bilaterality was related to fluid ability in older age (~71 and up). Besides illustrating that the optimal pattern of brain activity associated with good cognition appears to vary with age, based on these findings we tentatively suggest that later middle-age may represent a point where deviations from maintenance-like patterns of lateralization occur more frequently, but are not yet positively related to cognitive function. A key difference between the categorical and continuous age models is that left-lateralization was related to better fluid ability throughout young adulthood in the continuous age models, whereas this effect did not quite reach significance for younger adults in the categorical age model. Only a small number of younger adults displayed strong prefrontal bilaterality here, and it seems plausible that may have limited our ability to observe a robust lateralization effect in this age group relative to when the effect was estimated in a continuous model across the full sample. Additionally, we note that the sample sizes differed considerably between younger (*n* = 90), middle-aged (*n* = 124), and older adults (*n* = 244), which likely led to older age groups having somewhat increased statistical power; however, even the younger adult age group was relatively large and had suitable sensitivity (>0.8) to observe a medium-sized effect (*r* = 0.3, G*Power ver. 3.1). Another potential limitation in our aging analysis is that we used an MNI template that was developed in a largely young to middle-aged sample (ICBM152 range: 18–44y). This could have led to increased error in estimation of laterality terms for older adults, though we did see that laterality effects in older adults were replicated across a wide range of analyses and the low / high demand blocks.

We note that the crossover effect of left-lateralization being positively related to cognition in middle age but negatively related to cognition in older age appears to represent a developmental discontinuity. The STAC model (Park and Reuter-Lorenz, 2009; Reuter-Lorenz and Park, 2014) predicts such a discontinuity and suggests that lateralization differences in middle-aged and older adults reflect individual differences in the developmental timing of compensatory brain activity. More specifically, the STAC model predicts that middle-aged adults are most successful when they show youthful brain function, and that a compensatory shift to bilaterality will occur at some point in the lifespan, but the later the better (Park and Reuter-Lorenz, 2009). The model specifies that scaffolding is a response to neural insults, thus middle-aged adults who show this pattern typical of older adults likely have some latent pathology, such as early prefrontal cortical thinning (Raz et al., 2010), that makes them more likely to become cognitive decliners over time. Indeed, we observed here that thinner left prefrontal cortex in middle-aged adults was related to more bilateral prefrontal recruitment, likely as an attempt to compensate.

The present findings illustrate the importance of studying middle-aged adults to understand the mechanisms underlying optimal cognitive aging. The findings suggest that there is significant variability in both the timing and pattern of lateralization of brain activity during semantic retrieval that may reveal evidence of brain health or subtle early pathology. The positive association between bilaterality and cognitive function in older adults, but not middle-aged adults, may provide a window into a future cognitive trajectory. We note that longitudinal data are needed to provide more conclusive evidence for a stage-based model of changing brain activation patterns associated with optimal cognitive aging and to delineate whether differences in lateralization primarily represent an attempt to compensate for declining brain structure, as the STAC model suggests, or reflect increased brain activity due to lower cognitive ability that has both a heritable and genetic component. In addition, examination of other fMRI and cognitive tasks in existing studies with large lifespan samples will be useful in determining the generality of the present findings, and to clarify what characterizes a healthy brain at different ages and what patterns may signal risk and a need for potential intervention.

## Figures and Tables

**Table 1:** Demographic and descriptive statistics.

	Younger Adults (*n* = 90) Mean (SD)	Middle-Aged *(n* = 127)	Older Adults (*n* = 244)
Age (y)	29.19 (5.62)	51.61 (5.45)	72.48 (7.65)
Age Range (y)	20–39	40–59	60–89
Male/Female (% Female)	34/56 (62.2%)	47/80 (63.0%)	95/149 (61.1%)
PFC Laterality, low demand^2^	0.43 (0.37)	0.36 (0.37)	0.28 (0.40)
PFC Laterality, high demand	0.45 (0.33)	0.43 (0.33)	0.37 (0.36)
VLPFC Laterality, low demand^2^	0.51 (0.37)	0.49 (0.36)	0.40 (0.40)
VLPFC Laterality, high demand	0.54 (0.31)	0.56 (0.30)	0.49 (0.35)
DLPFC Laterality, low demand^2^	0.29 (0.42)	0.19 (0.43)	0.15 (0.41)
DLPFC Laterality, high demand	0.29 (0.38)	0.27 (0.38)	0.24 (0.39)
Fluid Ability (Z-score)^1,2,3^	0.98 (0.63)	0.37 (0.92)	−0.55 (0.76)
Processing Speed (Z-score)^1,2,3^	1.04 (0.76)	0.30 (0.78)	−0.54 (0.79)
Working Memory (Z-score)^1,2,3^	0.66 (0.90)	0.31 (1.09)	−0.41 (0.78)
Reasoning (Z-score)^1,2,3^	0.81 (0.62)	0.32 (0.91)	−0.47 (0.89)
Crystalized Ability (Z-score)^1,2^	−0.50 (0.92)	−0.02 (1.11)	0.19 (0.90)
Episodic Memory (Z-score)^1,2,3^	0.59 (0.99)	0.23 (0.96)	−0.33 (0.89)
Judgment RT (low demand)^2,3^	929 (125)	940 (143)	1007 (142)
Judgment RT (high demand)^3^	1207 (192)	1207 (174)	1268 (178)
L VLPFC/DLPFC Thickness^1,2,3^	2.63 (0.20)	2.53 (0.21)	2.36 (0.20)
R VLPFC/DLPFC Thickness^1,2,3^,	2.67 (0.18)	2.51 (0.22)	2.34 (0.20)

*Note*. Laterality index scores closer to 1 indicate stronger left-lateralization and scores closer to 0 reflect more bilateral recruitment; scores were reported for both levels of semantic judgment demand for the combined ventrolateral/dorsolateral prefrontal mask (PFC), as well as individual regions. Z-scores computed from full sample. Between-subjects ANOVAs revealed a main effect of age group for all cognition measures, laterality for the low demand condition, median RTs for both conditions, and cortical thickness in both the left and right prefrontal ROIs (all *p*’s < 0.01). Abbreviations: DLPFC, dorsolateral prefrontal cortex; VLPFC, ventrolateral prefrontal cortex; RT, response time. Significant differences from Tukey’s HSD test are reported between.

1 younger and middle-aged adults,

2 younger and older adults,

3 middle-aged and older adults.

**Table 2:** Univariate activations for primary prefrontal regions of interest by age.

	Younger Adults (n = 90)	Middle-Aged (n = 127)	Older Adults (n = 244)
Region	Mean (SD)		
**Low Demand Condition**			
L PFC (VLPFC/DLPFC)	.14 (.23)***	.13 (.23)***	.23 (.24)***
R PFC (VLPFC/DLPFC)	−.04 (.20)	−.002 (.22)	.09 (.22)***
L VLPFC	.29 (.27)***	.26 (.28)***	.35 (.27)***
R VLPFC	.002 (.24)	.01 (.26)	.10 (.27)***
L DLPFC	.03 (.23)	.03 (.23)	.13 (.23)***
R DLPFC	−.06 (.21)**	−.02 (.22)	.08 (.22)***
**High Demand Condition**			
L PFC (VLPFC/DLPFC)	.40 (.28)***	.38 (.26)***	.41 (.26)***
R PFC (VLPFC/DLPFC)	.12 (.22)***	.13 (.24)***	.17 (.24)***
L VLPFC	.60 (.34)***	.55 (.30)***	.58 (.31)***
R VLPFC	.14 (.26)***	.14 (.27)***	.17 (.29)***
L DLPFC	.26 (.27)***	.26 (.26)***	.28 (.26)***
R DLPFC	.10 (.23)***	.12 (.25)***	.16 (.25)***

*Note*. Raw beta means (task – fixation) for each age group are presented for each primary prefrontal region and for both semantic judgment conditions (low and high demand). Abbreviations: DLPFC, dorsolateral prefrontal cortex; VLPFC, ventrolateral prefrontal cortex. Two-tailed p-values:

* *p* < .05,

** *p* < .01,

*** *p* < .001.

**Table 3:** Regression models using age and prefrontal laterality to predict fluid ability.

Model	Regressors	Single regressor statistics		
		*b* values	*t* scores	*p* values
Categorical Age	Intercept	0.22	3.15	.002
	Sex	0.21	3.34	.001
	Crystallized Ability	0.40	12.43	< 0.001
	Age (young - middle)	0.80	8.39	< 0.001
	Age (old - middle)	−1.00	−13.66	< 0.001
	Age (old - young)	−1.80	−20.62	< 0.001
	Laterality	0.18	2.98	.003
	Age × Laterality (young - middle)	−0.07	−0.76	.446
	Age × Laterality (old - middle)	−0.30	−4.00	< 0.001
	Age × Laterality (old - young)	−0.22	−2.62	0.009
Continuous Age	Intercept	−0.10	−1.88	.061
	Sex	0.21	3.60	< 0.001
	Crystallized Ability	0.39	13.47	< 0.001
	Age_linear_	−0.93	−14.05	< 0.001
	Age_quadratic_	−0.01	−0.31	756
	Age_cubic_	0.07	2.37	.018
	Laterality	0.02	0.40	.688
	Age_linear_ × Laterality	−0.15	−2.25	.025
	Age_quadratic_ × Laterality	−0.003	−0.08	.937
	Age_cubic_ × Laterality	0.03	0.87	.386

*Note*. All regressors except age group and sex were entered as standardized variables and have b-values that are standardized *β*-values. Laterality was measured in the combined ventrolateral/dorsolateral prefrontal mask. Terms comparing old vs. young estimated from separate models.

## Data Availability

Derived data and analysis code for this project are accessible at: https://osf.io/vr6qj/? and https://identifiers.org/neurovault.collection:13143. Software used to process these data are freely available online as detailed in the Material and methods section. The LI-tool toolbox is freely available at: http://www.medizin.unituebingen.de/kinder/en/research/neuroimaging/software/.

## Footnotes

2 A similar pattern of results was observed using a weighted laterality index.

3 In a separate regression where crystallized ability was not included, a significant age group x lateralization effect was still observed, F(1, 456) = 8.74, p < .001, with a pattern of results similar to Figure 4c.
